# Machine Learning Identifies Six Genetic Variants and Alterations in the Heart Atrial Appendage as Key Contributors to PD Risk Predictivity

**DOI:** 10.3389/fgene.2021.785436

**Published:** 2022-01-03

**Authors:** Daniel Ho, William Schierding, Sophie L. Farrow, Antony A. Cooper, Andreas W. Kempa-Liehr, Justin M. O’Sullivan

**Affiliations:** ^1^ Liggins Institute, The University of Auckland, Auckland, New Zealand; ^2^ MRC Lifecourse Epidemiology Unit, University of Southampton, Southampton, United Kingdom; ^3^ Australian Parkinsons Mission, Garvan Institute of Medical Research, Sydney, NSW, Australia; ^4^ St Vincent’s Clinical School, UNSW Sydney, Sydney, NSW, Australia; ^5^ Department of Engineering Science, The University of Auckland, Auckland, New Zealand; ^6^ Brain Research New Zealand, The University of Auckland, Auckland, New Zealand; ^7^ The Maurice Wilkins Centre, The University of Auckland, Auckland, New Zealand

**Keywords:** Parkinson’s disease, heart atrial appendage, SNCA, PD-SNPs, tissue specific eQTL, machine leaning, GBA, Brain Cerebellum

## Abstract

Parkinson’s disease (PD) is a complex neurodegenerative disease with a range of causes and clinical presentations. Over 76 genetic loci (comprising 90 SNPs) have been associated with PD by the most recent GWAS meta-analysis. Most of these PD-associated variants are located in non-coding regions of the genome and it is difficult to understand what they are doing and how they contribute to the aetiology of PD. We hypothesised that PD-associated genetic variants modulate disease risk through tissue-specific expression quantitative trait loci (eQTL) effects. We developed and validated a machine learning approach that integrated tissue-specific eQTL data on known PD-associated genetic variants with PD case and control genotypes from the Wellcome Trust Case Control Consortium. In so doing, our analysis ranked the tissue-specific transcription effects for PD-associated genetic variants and estimated their relative contributions to PD risk. We identified roles for SNPs that are connected with *INPP5P*, *CNTN1*, *GBA* and *SNCA* in PD. Ranking the variants and tissue-specific eQTL effects contributing most to the machine learning model suggested a key role in the risk of developing PD for two variants (rs7617877 and rs6808178) and eQTL associated transcriptional changes of *EAF1-AS1* within the heart atrial appendage. Similarly, effects associated with eQTLs located within the Brain Cerebellum were also recognized to confer major PD risk. These findings were replicated in two additional, independent cohorts (the UK Biobank, and NeuroX) and thus warrant further mechanistic investigations to determine if these transcriptional changes could act as early contributors to PD risk and disease development.

## Introduction

Parkinson’s disease (PD) is a complex neurodegenerative disease with a range of causes and clinical presentations. The diagnosis of PD is based on the presence of the cardinal motor symptoms (bradykinesia; muscular rigidity; 4–6 Hz resting tremor; postural instability) (Clarke et al., 2016). Genome wide association studies (GWAS) have identified human genetic variants that are associated with the risk of developing PD (Spencer et al., 2011; Nalls et al., 2019). In the most recent PD GWAS meta-analysis, Nalls et al. (2019) identified 90 independent single nucleotide polymorphisms (SNPs) that are significantly associated with PD risk. There are an additional 290 PD-associated GWAS SNPs (279 in non-coding and 11 in coding regions) listed in the GWAS catalog. However, it is difficult to understand how these variants confer PD risk because the majority of the PD SNPs are located in non-coding regions of the genome (Visscher et al., 2012, 2017; Farrow et al., 2021).

Non-coding SNPs have been shown to be enriched at regulatory loci and can act as expression quantitative trait loci (eQTLs) (Duggal et al., 2014; Fadason et al., 2017, 2018; Delaneau et al., 2019; Yu et al., 2019). eQTLs typically explain a fraction of the variation in mRNA expression levels for target genes, either in *cis* (<1 Mb apart in the linear sequence) or *trans* (>1 Mb apart or located on a different chromosome). Regulatory variants (i.e., eQTLs) can impact different genes in different tissues, making it challenging to determine how SNPs convey risk for a phenotype. Determining the relative contributions of the eQTLs to the risk of developing a disease would help identify the eQTL-gene-tissue combinations that convey the risk associated with the variant (Ho et al., 2021). We have demonstrated that the three-dimensional structure of the genome can be used to help identify eQTL-gene pairs and thus the biological pathways that putatively contribute to disease etiology (Aguet et al., 2017; Schierding et al., 2020). Yet, approaches that calculate relative estimates of the tissue specific contributions that SNPs make to disease development remain elusive.

We reasoned that if PD-associated SNPs contribute to disease development through gene regulatory effects, then the tissue-specificity of these eQTLs may be an important consideration for the aetiology of the disease (Aguet et al., 2017; Ongen et al., 2017; Ho et al., 2021). Therefore, we developed a machine-learning predictor model for PD disease status that utilises and selects SNPs (without eQTLs in GTEx) and tissue-specific eQTL data, for case and control cohorts, to reveal the tissue-specific regulatory effects that are associated with PD risk. Briefly, we used a matrix of: 1) PD-associated SNPs that act as eQTLs, 2) the genes regulated by these eQTLs; 3) the tissues in which the eQTL effects were observed; and 4) SNPs that do not have eQTLs in GTEx to build a logistic predictor that was validated using genotype data from three independent studies (Spencer et al., 2011; Nalls et al., 2014; Bycroft et al., 2018). The logistic predictor model that had the highest PD predictive ability, was trained and selected using the Wellcome Trust Case Control Consortium (WTCCC) cohort. The predictor model was then validated using two datasets derived from the UK Biobank (Bycroft et al., 2018) and NeuroX-dbGap (Nalls et al., 2014). Our predictor ranked the relative contributions that six non-eQTL PD SNPs, and eQTLs that modulated gene regulation specifically within the heart atrial appendage as making the largest contributions to PD risk development.

## Methods

**FIGURE 1:**
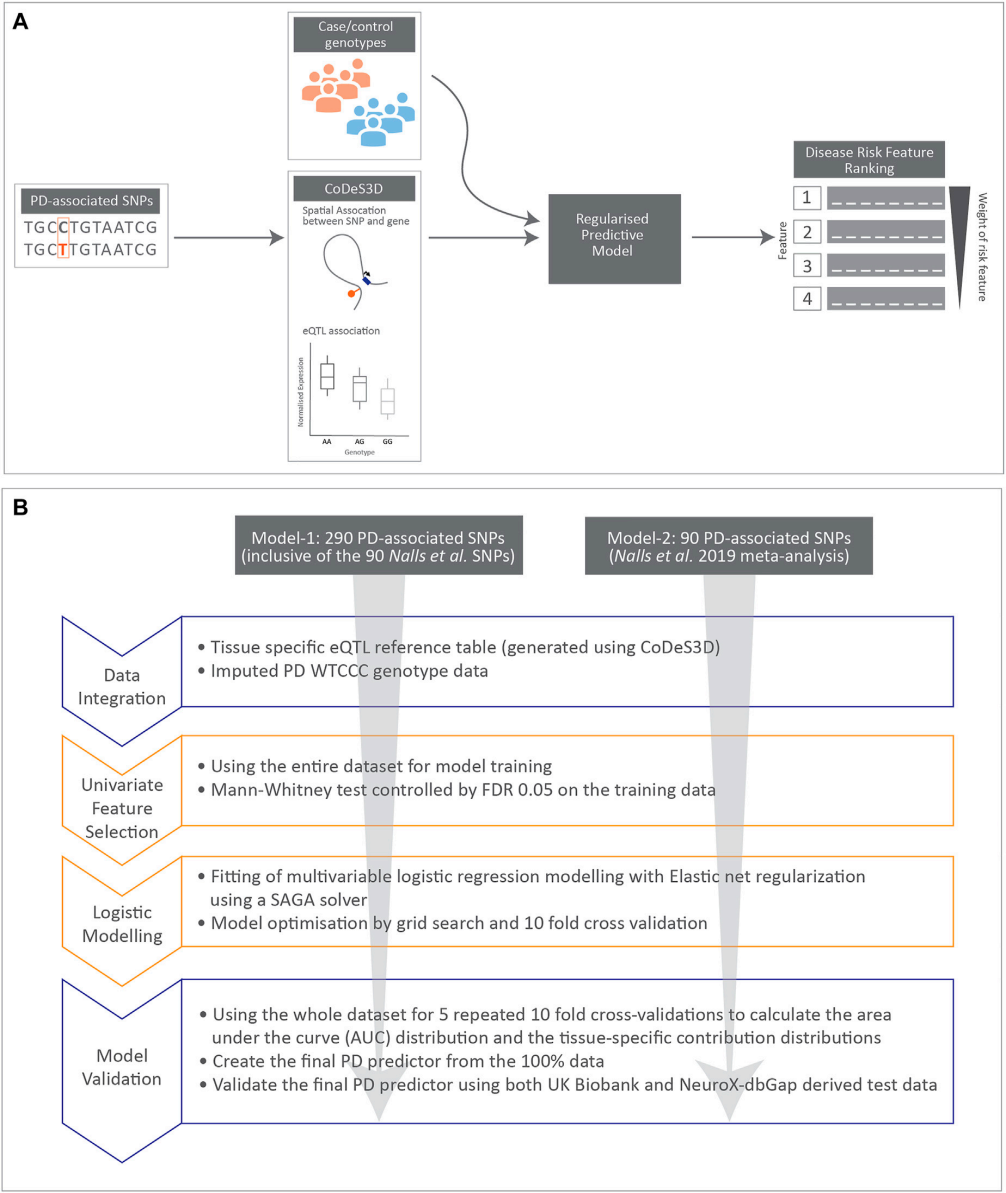
Cartoon illustrating data integration and workflow for regularised logistic regression modelling undertaken in this manuscript. **(A)** Schematic diagram for data integration used to rank disease risk features. **(B)** Workflow used to create the two regularised logistic regression predictor models for PD.

Workflow for developing the PD predictor model-1 and -2 (Figure 1).

### Generation of Tissue Specific PD eQTL Reference Table

GWAS SNPs associated with PD (*n* = 290, *p-*value < 1.0 × 10^−5^; Supplementary Table S1) were obtained from the GWAS catalogue (www.ebi.ac.uk/gwas, downloaded August 27, 2020). This SNP set included young adult-onset Parkinsonism SNPs (Siitonen et al., 2017) and the 90 SNPs identified by the most recent meta-analysis by Nalls et al. (2019). The PD associated SNPs were analysed by CoDeS3D and mapped to their tissue-specific eQTL effects for creating a PD eQTL reference table (Supplementary Material).

### WTCCC Cohort Cleaning and Genotype Imputation

The PD genotype dataset was acquired from the WTCCC (Request ID 10584) and were imputed by Sanger imputation service (https://imputation.sanger.ac.uk) (Supplementary Material).

### Creation of a Weighted WTCCC PD Genotype eQTL Effect Matrix

We created a matrix that combined individual genotypes with the eQTL effects for the PD-associated SNPs (Supplementary Material) which contains three groups of data fields:

1. Individual sample information
2. Individual sample PD-associated SNP genotype (SNP minor-allele count) weighted by GTEx tissue-specific eQTL normalised effect sizes (NES)
3. Individual PD-associated SNP genotype for the SNPs without known eQTL effects

### Generation, Training, and Validation of the Regularised Logistic Regression Models (Model-1 and Model-2)

We created two regularised logistic regression models (see below): for model-1 from a weighted WTCCC PD genotype eQTL matrix for all 290 SNPs (GWAS catalogue) and for model-2 from a weighted WTCCC PD genotype eQTL matrix for the subset of 90 SNPs (Nalls et al., 2019).

We developed a regularised logistic regression predictor that incorporated a: 1) Mann-Whitney *U* tests in combination with Benjamini-Yekutieli (BY) procedure for controlling False Discovery Rate (FDR); and 2) multivariate prediction step with regularization that considers all features in context and removes redundant information, to identify the best combination of features for prediction of PD.

### Calculation of Tissue-Specific Contributions to PD Risk

The 50 PD regularised logistic regression predictors created from the five repeats of 10-fold cross-validation were used to test the predictive power of the models created with the optimised predictor hyperparameters. Tissue-specific contributions to the PD risk were extracted from each of the 50 PD regularised logistic regression predictors as the sum of the absolute values of the model weights associated with each tissue.

### Validation of Model-1 and Model-2

The generalising PD predictive power of models-1 and -2 was validated by testing on two independent test datasets derived from the UK Biobank (30 test cohorts) and NeuroX-dbGap genotype data (Supplementary Material).

### Data Analysis

All statistical tests were performed with Scikit-learn (version 0.23.2) (Abraham et al., 2014), and tsfresh (version 0.16.0) (Christ et al., 2018). Polygenic Risk Scores were calculated by R (version 3.2.3) with pROC library (Robin et al., 2011; R Core Team, 2014).

## Results

### PD-Associated SNPs Are Tissue Specific eQTLs for 1,334 eGenes

We hypothesised that PD SNPs modulate disease risk through tissue-specific eQTL effects (i.e., eQTL-eGene) (Aguet et al., 2017; Ongen et al., 2017). We analysed 290 PD-associated GWAS SNPs (Supplementary Table S1) for spatial eQTL interactions (Ramani et al., 2016; Fadason et al., 2017; Pal et al., 2019) across 49 GTEx tissues (Aguet et al., 2017). 231 of the 290 (79.7%) PD SNPs tested were involved in 18,041 tissue-specific eQTL associations (Benjamini–Hochberg FDR < 0.05 (Benjamini and Hochberg, 1995); Supplementary Table S2), regulating 1,334 eGenes across the 49 GTEx tissues. Gene ontology analysis (David Functional Annotation) (Jiao et al., 2012) identified that the regulated genes were significantly enriched for intracellular signal transduction, antigen processing and presentation of peptides, among other pathways (Supplementary Table S3).

### Modelling Genotype Data to Identify the Genetic Risk Associated With Tissue-Specific eQTL Effects for PD Disease Status

Understanding the impacts and complex networks associated with eQTLs is challenging. We hypothesised that regularised logistic regression models could be used to identify and rank the tissue-specific eQTLs that were significant contributors to PD risk.

We integrated the CoDeS3D eQTL analysis of the 290 PD SNPs with the genotype data for individuals within the WTCCC(Burton et al., 2007) PD cohort (4,366 individual samples: 1,698 cases and 2,668 controls; methods) (Spencer et al., 2011). Of the 290 PD SNPs, 281 SNPs were present in the WTCCC data. This resulted in the generation of a PD-SNP derived weighted WTCCC PD genotype eQTL effect matrix containing 17,829 tissue-specific eQTL-eGene pairs (227 SNPs, 1,310 eGenes, 49 tissues) and 54 (of the 281) SNPs that had no known eQTL effects following our CoDeS3D analysis. Uninformative features for PD prediction were removed using a Mann-Whitney *U* test (McKnight and Najab, 2010) (FDR <0.05) (Methods). After filtering, 11,288 PD SNP derived features (53 SNPs, 245 eGenes, 49 tissues) remained within the relevant attribute subset of the weighted WTCCC PD genotype eQTL effect matrix.

To test the effectiveness of the Mann-Whitney *U* test filter, we generated a PD and type 1 diabetes (T1D) SNP derived eQTL effect matrix using a mixed set of 290 PD and 313 T1D-associated SNPs and integrating with the WTCCC PD cohort genotypes (Supplementary Table S4). The PD + T1D SNP derived tissue-specific eQTL effect matrix included 25,052 SNP related data fields (556 SNPs, 1927 eGenes, 49 tissues). After the Mann-Whitney *U* test filtering (FDR <0.05), 11,147 of the data fields (45 SNPs, 209 eGenes, 49 tissues) were selected using PD as the phenotypic outcome. Only one of the 313 (0.32%) T1D-associated SNP, rs1052553, remained following the Mann-Whitney *U* test filtering. Although rs1052553 has not previously been associated with PD in GWA studies, it has been implicated in PD as part of a PD risk haplotype (Tobin et al., 2008; Wider et al., 2010). Therefore, these results confirm that the Mann-Whitney *U* test filters uninformative data while preserving valuable PD information for our modelling.

We created regularised logistic regression models for PD risk using the Mann-Whitney *U* test filtered PD variant derived eQTL effect matrix (11,288 PD-SNP derived features [53 SNPs, 245 eGenes, 49 tissues]). The AUCs of the 50 PD regularised logistic regression predictors had a mean of 0.565 (distributed from 0.516 to 0.637) and a standard deviation of 0.024 (generated with the optimised predictor model hyperparameters by five repeats of 10-fold cross validation). The final PD predictor model (model-1) was trained using the entire WTCCC PD cohort. After the Mann-Whitney *U* test filtered WTCCC PD variant derived eQTL effect matrix contained 17,829 variant derived features. Model-1 selected 827 tissue-specific eQTLs and six SNPs with no eQTL effect (Supplementary Table S5). Model-1 had an enhanced diagnostic ability as represented by an AUC of 0.627 obtained using the training data.

We validated the predictive power of model-1 using two independent PD cohorts (UK Biobank (Bycroft et al., 2018) (30 datasets of 923 cases and 1,456 controls) and NeuroX-dbGap (Nalls et al., 2014, 2015)). Model-1 was validated in both cohorts, producing mean AUCs of 0.572 and 0.571 in the UK BioBank and NeuroX-dbGap cohorts, respectively. These two validation results are highly consistent and within the range of the model AUCs (0.516–0.637) estimated by the 50 optimised logistic regression predictor models.

### eQTLs Specific to the Heart Atrial Appendage Contribute to Genetic Risk in PD

We used the magnitude of the model weights (coefficients) for the genetic features, grouped by tissue-specificity of the effects, in the logistic regression model-1 as proxies for the contribution of the features to PD risk.

**FIGURE 2:**
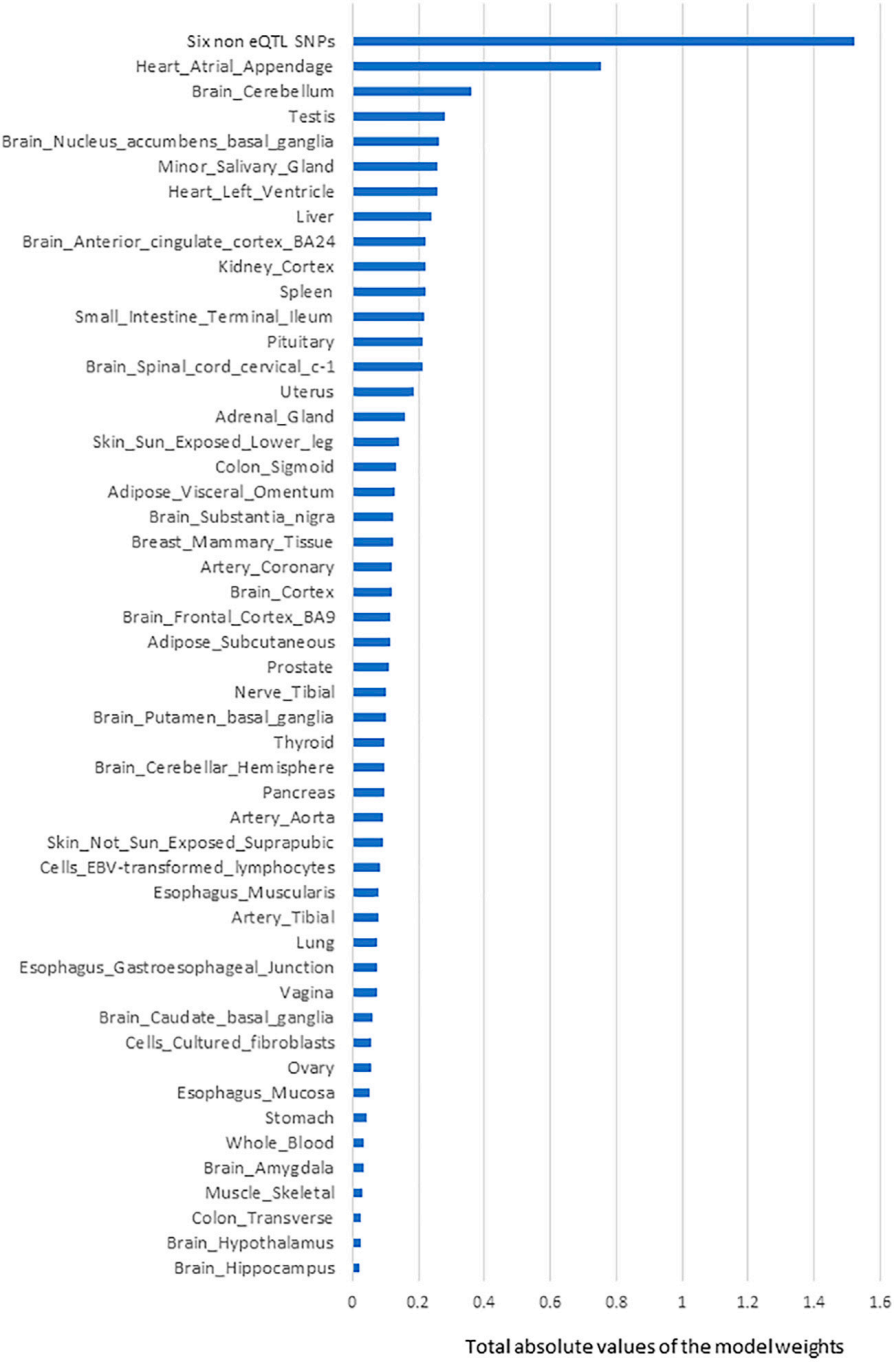
The rank order of tissue-specific risk contributions to risk of developing PD calculated using model-1. Tissue PD risk contributions were the sum of the absolute values of the model weights (coefficients) of the features used in the logistic regression predictor (model-1) according to their tissues. The SNPs/eQTLs that contributed to each category are listed (Supplementary Table S5).

Six SNPs that had no identified eQTL effects (from CoDeS3D analysis of GTEx) made the most significant group contribution (18% of the total model weight) to the risk of PD development (Table 1; Figure 2). The six non-eQTL SNPs are: rs117896735, rs144210190, rs35749011, rs12726330, rs356220 and rs5019538 (Table 1). Note that the GTEx study (Aguet et al., 2017) removed rs356220 and rs5019538 from the tissue-specific eQTL data as part of their QC processing. Therefore, we were unable to test if rs356220 and rs5019538 were eQTLs. rs117896735 also has no eQTL effect information found in GTEx database. The other three SNPs (rs144210190, rs35749011 and rs12726330) were not detected by CoDeS3D to have spatial eQTL and eGene interactions within the Hi-C libraries used in this study.

**TABLE 1:** SNPs identified as being the main contributors to model-1. a) SNPs with no detected eQTL effects, and b) eQTL effects within the Heart Atrial Appendage. The model weight is the coefficient assigned to each variant or eQTL in the logistic regression predictor model-1. “*” indicates the non eQTL SNP is in the 90 SNPs of Nalls et al.

a)	
SNP (no detected eQTLs)	Model weight
*rs117896735_A	0.42436
rs1442190_A	0.40106
*rs35749011_A	0.24949
rs12726330_A	0.18701
rs356220_T	0.17507
*rs5019538_G	−0.08418

b)			
Tissue	eQTL (rsID_major allele)	Gene	Model weight
Heart_Atrial_Appendage	rs7617877_A	EAF1-AS1	0.28996
Heart_Atrial_Appendage	rs6808178_T	EAF1-AS1	0.25261
Heart_Atrial_Appendage	rs6808178_T	TMEM161B-AS1	0.16339
Heart_Atrial_Appendage	rs11707416_A	P2RY12	0.01163
Heart_Atrial_Appendage	rs26431_G	EIF3KP1	0.0089
Heart_Atrial_Appendage	rs17577094_G	RP11-259G18.3	0.00703
Heart_Atrial_Appendage	rs365825_G	RP11-259G18.3	−0.00467
Heart_Atrial_Appendage	rs17577094_G	LRRC37A4P	−0.00434
Heart_Atrial_Appendage	rs17577094_G	KANSL1-AS1	0.0036
Heart_Atrial_Appendage	rs8070723_G	RP11-259G18.3	−0.00294
Heart_Atrial_Appendage	rs365825_G	LRRC37A4P	0.00237
Heart_Atrial_Appendage	rs365825_G	KANSL1-AS1	−0.00128
Heart_Atrial_Appendage	rs17577094_G	DND1P1	0.00116
Heart_Atrial_Appendage	rs17577094_G	MAPK8IP1P2	0.00058
Heart_Atrial_Appendage	rs199515_G	RP11-259G18.3	−0.00011

For the top six contributing SNPs to the model, our analyses did not identify any spatial eQTL interactions. The SNPs that are in high linkage disequilibrium (r2 > 0.8) with these six SNPs also did not have significant spatial eQTLs. However, previous research has shown connections between these SNPs and three well-known PD-associated genes (*INPP5F*, *GBA*, *SNCA*) (Siddiqui et al., 2016; Berge-Seidl et al., 2017; Riboldi and Di Fonzo, 2019; Cao et al., 2020), and an additional gene (*CNTN1*). rs117896735, the top contributor to model-1, is an intronic variant of *INPP5F* and has previously been identified as eQTL for *INPP5F* transcript levels (the IPDGC locus browser (Grenn et al., 2020)).

**FIGURE 3:**
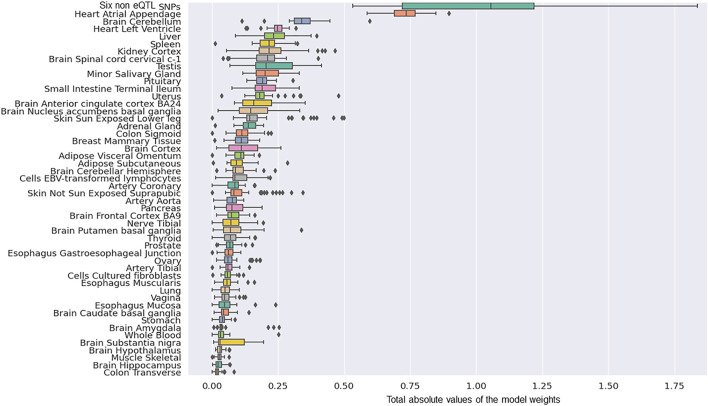
The rank order of tissue-specific risk contributions calculated across 50 predictor models created from randomised modelling and model-1’s hyperparameters. The tissue ranking was consistent with that observed for model-1.

The next most significant contributions to the risk of PD development involved eQTLs that affected the Heart Atrial Appendage (9%) and Brain Cerebellum (4%; Figure 2). The substantia nigra is viewed as a central brain region in PD yet eQTL gene regulation specific to the substantia nigra contributed ∼1.5% of the risk of PD development. We repeated the calculation of the tissue-specific contribution ranking using data from the 50 optimised predictor models, generated with model-1’s hyperparameters by five repeats of 10-fold cross validation (randomizing the full Mann-Whitney *U* test filtered PD variant derived eQTL effect matrix),. Again, SNPs lacking known eQTL effects, Heart Atrial Appendage, and Brain Cerebellum were identified as the top three genetic contributors to the risk of PD development (Figure 3).

Fifteen eQTLs contributed to the Heart Atrial Appendages contribution to the risk of developing PD measured in model-1 (Table 1). Notably, the two biggest eQTL contributors, rs7617877 and rs6808178, each accounted for approximately 3% of the total model weight. rs7617877 and rs6808178 are in high linkage disequilibrium (*R* ^2^ = 0.86) (Machiela and Chanock, 2015) within European populations. rs7617877 and rs6808178 do not show detectable spatial regulatory associations with their nearest genes and instead both act as eQTLs for a gene >13 Mb downstream, *EAF1-AS1*, in the Heart Atrial Appendage. *EAF1-AS1* is a long antisense non-coding RNA gene transcribed in antisense to *EAF1*, that undergoes an isoform switch, and has a significantly different transcript usage in the brains of patients with Parkinson’s disease (Dick et al., 2020). Interestingly, rs6808178 also acts as a Heart Atrial Appendage eQTL for *TMEM161B-AS1* (Table 1), which has also been implicated in neurodegeneration (Boros et al., 2020).

### Creating a PD Logistic Regression Predictor Model Using the 90 SNPs From the PRS Calculated by Nalls et al.

In the latest PD GWAS meta-analysis, Nalls et al. (2019) identified 90 SNPs that contribute to a PRS model for PD risk. We therefore sought to understand the PD risk contribution that was specific to these 90 SNPs and created a logistic regression predictor model using only this subset. 88 of the 90 variants passed quality control (post-imputation data cleaning and quality checking). The 88 SNPs were integrated with the WTCCC PD genotype data to create a PD SNP derived eQTL effect matrix of WTCCC individual samples (4,366 individual samples: 1,698 cases and 2,668 controls). The PD SNP derived eQTL effect matrix contained 3,206 features consisting of related tissue-specific eQTL-eGene pairs (76 SNPs, 518 genes, 49 tissue types) and 12 SNPs that lacked CoDeS3D detectable eQTL effects. Mann-Whitney *U* test filtering (FDR < 0.05) left 920 features (12 SNPs, 95 genes, 49 tissue types) that were used in the subsequent logistic regression modelling (Abraham et al., 2014). Model training was repeated using the optimised predictor hyperparameters and the eQTL effect matrix for the full WTCCC cohort to create predictor model-2. Model-2 achieved in-sample PD prediction with an AUC = 0.604 using 311 features (12 SNPs, 46 genes, 49 tissue types) (Supplementary Table S6) that included 308 tissue-specific eQTLs and three SNPs without known eQTL effects.

**FIGURE 4:**
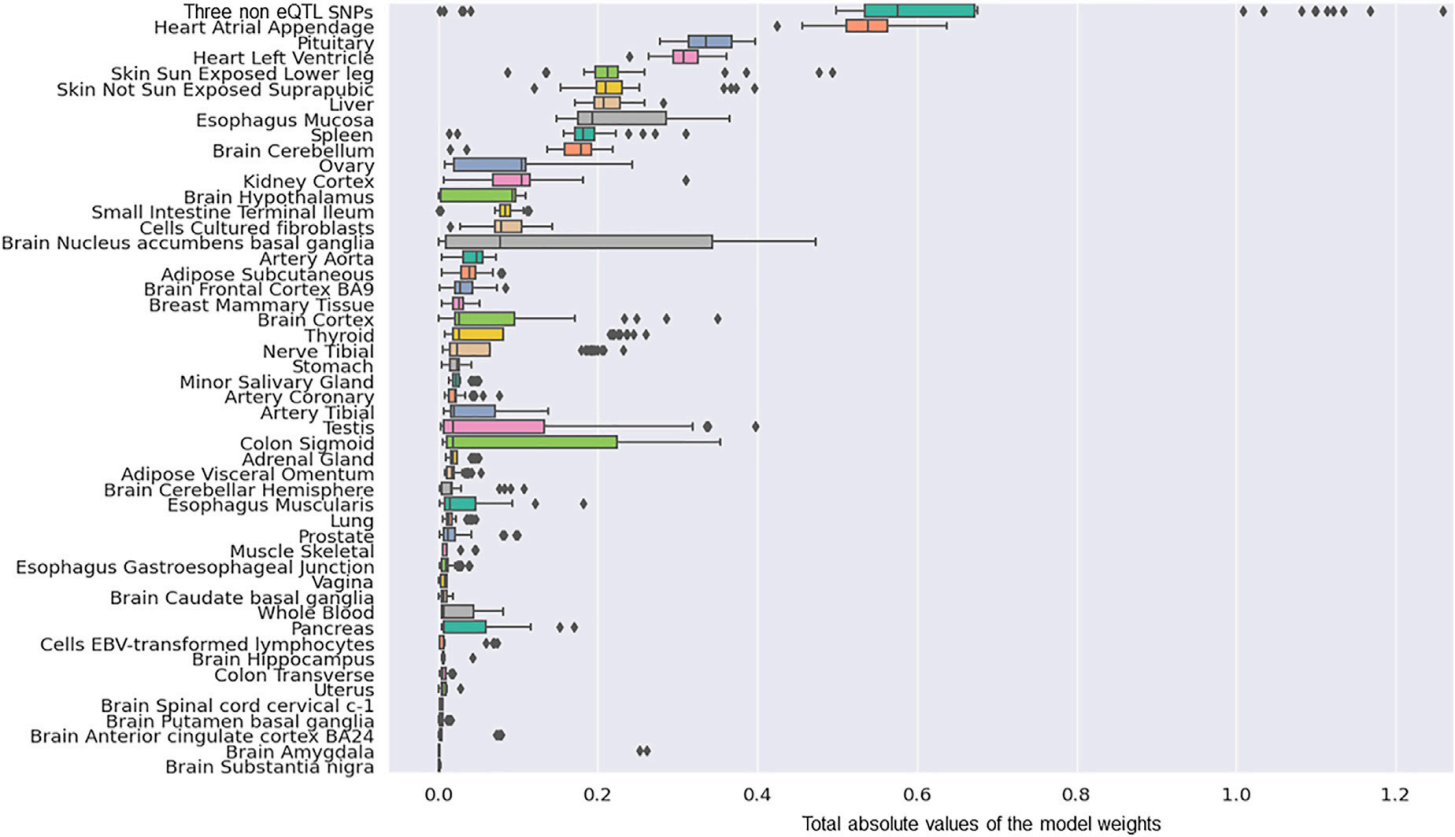
The group contributions of 50 predictors created with model 2 hyperparameters by five repeats of 10 fold cross-validation.

We determined the tissue-specific distribution for the 50 predictors that were created with model-2’s hyperparameters. The results we observed were consistent with what we observed using model-1 (Figure 4). Specifically, three SNPs (rs117896735, rs35749011 and rs5019538) with no identifiable eQTL effects (Table 2) and the eQTLs within the Heart Atrial Appendage were the top contributors to the risk of developing PD (Figure 4 and Table 2). The three non-eQTL SNPs appeared in both Model-1 and Model-2 and were observed to have similar effect sizes (both magnitude and direction) across both models. Also consistent with model-1, model-2 identified rs6808178 as the top eQTL contributing to the Heart Atrial Appendage signal.

**TABLE 2:** SNPs identified as being the main contributors to model-2. a) SNPs with no detected eQTL effects, and b) eQTL effects within the Heart Atrial Appendage. The model weight is the coefficient assigned to each variant or eQTL in the logistic regression predictor model-2.

a)	
SNPs (no detected eQTLs)	Model weight
rs117896735_A	0.54172
rs35749011_A	0.47224
rs5019538_G	−0.04028

b)			
Tissue	eQTL (rsID_major allele)	Gene	Model weight
Heart_Atrial_Appendage	rs6808178_T	EAF1-AS1	0.53154
Heart_Atrial_Appendage	rs26431_G	EIF3KP1	0.0079
Heart_Atrial_Appendage	rs504594_A	HLA-DQA2	−0.00333
Heart_Atrial_Appendage	rs62053943_T	RP11-259G18.3	−0.0014
Heart_Atrial_Appendage	rs62053943_T	DND1P1	−0.00092
Heart_Atrial_Appendage	rs62053943_T	KANSL1-AS1	−0.00064
Heart_Atrial_Appendage	rs62053943_T	LRRC37A4P	0.00061

The PRS using the 290 PD SNPs calculated for the WTCCC cohort (AUC = 0.634) was within the range of those calculated for model-1 (AUC = 0.516–0.637) using the weighted genotype eQTL matrix. Greater variation was observed for the 90 PD SNP PRS (AUC = 0.667) when compared to that calculated by model-2 (AUC range 0.504–0.631) using the weighted genotype eQTL matrix for the WTCCC cohort.

## Discussion

The mechanisms by which PD-associated genetic variants (Nalls et al., 2014; Escott-Price et al., 2015; Lill et al., 2015; Visscher et al., 2017) contribute to disease risk and development have not been fully elucidated. Yet, it is critical that we identify the mechanisms by which they impact on PD because this will allow patient stratification and the development of therapeutics that target disease progression and not just pathology. We used machine learning to understand the genetic architecture of PD risk, by identifying and ranking the pivotal risk variants and tissue-specific eQTL effects that contribute to such risk. Curated PD-associated SNPs from the GWAS catalogue (MacArthur et al., 2017) were analysed to identify their tissue-specific eQTL effects. Regularised logistic regression predictor models that evaluated PD risk were built and validated across three independent case:control cohorts (Spencer et al., 2011; Nalls et al., 2014; Bycroft et al., 2018). Model-1 (generated from 290 SNPs) identified six SNPs without known eQTL effects and the SNP modulated gene regulation within the Heart Atrial Appendage as being the major contributors to the predicted risk of developing PD. A second model (Model-2) that was generated using only 90 SNPs (Nalls et al., 2019) (which were previously identified to have the greatest predictive power with a PRS analysis) confirmed a subset of the top predictors we observed with model-1. Collectively, our results confirm roles for SNPs that are significantly connected with *INPP5P*, *CNTN1*, *GBA* and *SNCA* in PD and separately suggest a key role for transcriptional changes within the heart atrial appendage in the risk of developing PD. Effects associated with eQTLs located within the Brain Cerebellum were also recognized to confer major PD risk in the more extensive model (model-1) consistent with current hypotheses suggesting the Brain Cerebellum plays a role in PD development (Wu and Hallett, 2013; Seidel et al., 2017; Riou et al., 2021).

*INPP5F* is a known risk gene for PD (Cao et al., 2020) that regulates STAT3 intracellular signalling pathways (Kim et al., 2014) and has functional roles in cardiac myocytes and axons (Zhu et al., 2009; Zou et al., 2015). rs1442190 is an intronic variant within *CNTN1*, a known risk gene for dementia with Lewy bodies (Guerreiro et al., 2018; Chatterjee et al., 2020) that encodes a cell adhesion protein, which is important for axon connections and nervous system development (Anderson et al., 2018). rs35749011 and rs12726330 are linked to the well-known PD-associated gene *GBA* (Berge-Seidl et al., 2017) through strong linkage disequilibrium connections (*R* ^2^ = 0.77 (Machiela and Chanock, 2015)) with rs2230288 (Berge-Seidl et al., 2017; Mata et al., 2017), a missense coding variant located within *GBA*. rs35749011 has eQTL effects on *GBA* gene identified by the IPDGC database (Grenn et al., 2020). The final two SNPs, rs356220 and rs5019538, are located downstream of *SNCA. SNCA* encodes α-synuclein, which is central to PD pathogenesis (Siddiqui et al., 2016). The IPDGC database (Grenn et al., 2020) indicates that rs5019538 has eQTL effects on *SNCA*. Notably, rs356220 had the strongest association to PD in the original WTCCC GWAS (Spencer et al., 2011). Therefore, there is sufficient evidence that has previously associated these six variants with PD through connections to PD risk genes.

### Allele Specific Regulatory Changes in the Heart Atrial Appendage Confer PD Risk

We identified that eQTLs specific to the heart atrial appendage make a reproducible and substantial (second highest) contribution to the risk of developing PD. The heart atrial appendage is a trigger site of atrial fibrillation (AF) (Di Biase et al., 2010) and highly associated with hypertension and stroke (Hart and Halperin, 2001; Stöllberger et al., 2003; Turagam et al., 2018; Du et al., 2020). Notably, none of the heart atrial appendage eQTLs we identified have been previously associated with cardiac health or atrial fibrillation by GWAS (GWAS catalog, *November 2, 2021*). However, the genes on the opposite strands to the two antisense genes (i.e., *TMEM161B-AS1 and KANSL1-AS1*) have been previously implicated in regulating cardiac rhythm with zebrafish model (i.e., *TMEM161B* (Koopman et al., 2021)) and congenital heart defects in humans (i.e., *KANSL1* (Koolen et al., 2016; León et al., 2017)). However, there is a growing body of research indicating a close relationship between cardiovascular health and PD development (Awerbuch and Sandyk, 1994; Ascherio and Tanner, 2009; Fang et al., 2018; Scorza et al., 2018; Hong et al., 2019; Potashkin et al., 2020). The eQTL rs11707416 and its regulated eGene P2RY12 have been implicated in the brain blood barrier maintenance functions of microglial cells (Andersen et al., 2021). Moreover, AF has been strongly related to early-stage PD (Hong et al., 2019). Moreover, Moon et al. identified that patients with PD have an increased risk of AF, with a threefold increased risk (HR: 3.06, 95% CI: 1.20–7.77) of AF in younger PD patients (age: 40–49 years) (Han et al., 2021). Observations of a cross-sectional PD patient cohort have identified abnormal blood flow patterns in brains (Teune et al., 2014) and it is argued that AF-associated perturbation of the brain blood supply networks promotes tissue inflammation and damage leading to PD pathogenesis (Junejo et al., 2020).

Amongst the 15 eQTL features that combined to make the Heart Atrial Appendage’s contribution to the risk of developing PD (Tables 1, 2), eQTL up-regulation of *EAF1-AS1* (a long non-coding mRNA) made the greatest contribution. *EAF1-AS1* has different isoforms some of which overlap *EAF1* and *COLQ* (collagen like tail subunit of asymmetric acetylcholinesterase). Elevated *EAF1-AS1* transcript levels have previously been identified by differential gene expression analyses in brain tissue samples from PD patients (Dick et al., 2020). It is interesting to speculate that the impact of this change is mediated through the interaction of *EAF1-AS1* with *EAF1*. Notably, *EAF1* has been associated with both neural development (Liu et al., 2013) and TGF-β signalling (Liu et al., 2017), which is a key pathway in many cardiac physiological processes (Yousefi et al., 2020). As such, the deregulation of *EAF1-AS1* might impact on cardiac health. However, the anti-sense overlap is limited to the 3′ UTR of *EAF1* (UCSC Genome browser GRCh38/hg38). Therefore, we propose that future studies should investigate the regulatory impacts of *EAF1-AS1* on *EAF1* and the consequences of alterations in expression levels on heart function and PD disease. We contend that understanding this relationship may help to decipher the complex interactions connecting cardiovascular fitness and PD pathogenesis.

Similar to our work, Li et al. (2019) used linkage disequilibrium score regression (LDSC) analysis (Finucane et al., 2015, 2018) to identify enrichments of PD risk signals in six GTEx (Aguet et al., 2017) central nervous system tissues. However, three subsequent studies using LDSC have failed to reproduce Li et al.’s results (Gagliano et al., 2016; Reynolds et al., 2019; Bryois et al., 2021). LDSC focuses on measuring the risk enrichment of genes uniquely expressed in each GTEx tissue (Finucane et al., 2015, 2018). By contrast, our model does not assume unique tissue expression. Rather, it identifies the risk associated with the PD-SNP, or the expression of all genes modulated specifically by PD SNPs in different or multiple GTEx tissues. We therefore hypothesise that the fact that Li et al. did not identify any signals in heart tissues is likely due to the differences in the assumptions underlying the methodologies.

### What are the Functions of the Six SNPs for Which We Identified No eQTLs?

It should not be ignored that Model-1 (generated from 290 SNPs) identified six SNPs without known eQTL effects as making the greatest contribution to PD risk. A subset of these SNPs (rs117896735, rs35749011 and rs5019538) were confirmed in Model-2. Given the contribution of these SNPs to the models, it is interesting to speculate on the function(s) of these SNPs with respect to PD risk. As noted earlier, several of the SNPs are connected to well-known PD-associated genes (*INPP5F*, *GBA*, *SNCA*) (Siddiqui et al., 2016; Berge-Seidl et al., 2017; Riboldi and Di Fonzo, 2019; Cao et al., 2020). It remains possible that these SNPs may be eQTLs for these genes at different developmental stages, or in tissues or cell types that are not represented in the datasets we used in this study. Consistent with this, the top contributor to model-1 is an intronic variant of *INPP5F* that has previously been identified as an eQTL for *INPP5F* transcript levels (the IPDGC locus browser (Grenn et al., 2020)). However, the inclusion of these SNPs in the models did not assume a functional impact on transcription. As such, the SNPs may impact on PD risk through processes or functions that: 1) do not require the formation of spatially constrained eQTls; 2) affect transcript levels by another mechanism (e.g., DNA methylation and protein-protein interactions) (Ryan and Matthews, 2005; Volkov et al., 2016); or 3) function through another as yet uncharacterized mechanism. While we are currently unable to further expand on the function(s) of these SNPs, the significant contributions they make to PD require further experimental investigation.

### What Are the Limitations of Our Study?

We acknowledge several limitations within our work. Firstly, our models were not generated for use in clinical screening and the predictivity is clearly insufficient for such applications. Rather, our objective was to construct models that enabled the determination of the relative SNP-gene-tissue contributions to PD risk in individuals, using recognized PD-associated SNPs identified in population level association studies. We also acknowledge that the individuals in the included datasets are predominantly of European descent, and thus the significance of our findings are limited to this ethnicity. One limitation that impacts the vast majority of PD research is the lack of consistency in diagnostic criteria from one cohort to the other, and our study is not exempt from this.

The limitations within our study do not detract from the strengths of our model which included the fact that contributing features were: 1) validated across three independent cohorts; 2) easily identifiable; and 3) consistently identified genomic regions that are unanimously recognised as being associated with PD (e.g., *SNCA*).

Our approach provides a significant advance over other previously reported methods. The novelty revolves around the ability of our method to: 1) rank the contributions that SNPs make to a phenotype through regulatory changes; 2) identify the tissues in which these changes are occurring; and 3) include effects from variants that do not have detectable eQTLs in the reference library that is used in the assay. Finally, the consistency between models and ability to filter extraneous SNPs (e.g., T1D eQTLs) out of the final predictor is another strength of this study. The higher predictive power observed for model-1 (Supplementary Table S7) may be explained by the observation that the final model included more features (827 vs 308). However, given that model-1 leveraged 290 PD-associated SNPs, the result also suggests that the 90 SNPs, originally identified as part of the Nalls et al. PRS analysis (Nalls et al., 2019), do in fact contain the major genetic components that are associated with the risk of developing PD. Therefore, while other genetic signals clearly remain to be identified, the finding that both models consistently identified the same SNPs and heart atrial appendage eQTLs as the top contributors to the risk of developing PD further confirms the significance of these observations.

### Conclusion

In conclusion, we applied machine learning algorithms to rank the pivotal variants and tissue-specific eQTL effects that may contribute to the risk of developing PD by integrating PD-associated SNPs with information on genome organisation, tissue-specific eQTLs and the genotypes of PD cases and controls. Across our two models we consistently identified the same SNPs and heart atrial appendage eQTLs, linked to *EAF1-AS1* regulation, as the top contributors to the risk of developing PD. It could be argued that the lack of significant findings in established PD tissues (e.g., the *substantia nigra*) indicates that our models did not identify the biologically significant variants. However, studies show that disease associated SNPs are enriched in enhancer elements (GTEx Consortium, 2021) and it is widely recognized that pathology does not necessarily equate to the root cause of the PD. Rather the etiology of PD, and other movement disorders, is consistent with life-long contributions from early developmental changes. As such, we contend that our results, which replicate across three independent biological cohorts, provide insights into the non-motor multi-tissue features/processes (non-motor PD) that collectively, or singularly contribute to an individual’s progression to motor symptoms (motor PD) with age (Mhyre et al., 2012; Schapira et al., 2017). Future experiments should test the putative tissue specific enhancer activities we have identified using luciferase enhancer assays within edited human cell-lines that are isogenic except for the change of interest. Validation of the biological significance of the tissue level processes could then be addressed using tissue organoids and humanized animal models. These analyses should be performed in parallel with prospective studies that include analyses of the ever-expanding datasets (pulse oxygen levels, heart rate and blood pressure) that are being collected by wearable BioActive devices (e.g., Galaxy watch, Fitbits) Validation of our findings will provide insights into high value therapies for the prevention or delay of PD development.

## Data Availability

The original contributions presented in the study are included in the article/Supplementary Material, further inquiries can be directed to the corresponding authors. The CoDeS3D pipeline is available at: https://github.com/Genome3d/codes3d-v2. The Python scripts and machine learning code used in this analysis are available at: https://github.com/Genome3d/PD_lg_predictor_analysis. Python version 3.7.3 was used for all the Python scripts. eQTL information from 49 human tissues were obtained from the Genotype-Tissue Expression database [GTEx] v8; www.gtexportal.org. PD genotype datasets were acquired from the: Wellcome Trust Case Control Consortium (Request ID 10584); UKBioBank (Application Number 61507); NeuroX-dbGap (dbGap project#98581-1).
