# Supplementary material for: Machine Learning Identifies Six Genetic Variants and Alterations in the Heart Atrial Appendage as Key Contributors to PD Risk Predictivity
Source: Front Genet. 2022 Jan 3;12:785436. doi: 10.3389/fgene.2021.785436 (PMC8762216; doi:10.3389/fgene.2021.785436)
Supplement: Supplementary file 8 [file DataSheet2.docx]

**List of Supplementary Tables for Machine learning identifies six genetic variants and alterations in the Heart Atrial Appendage as key contributors to PD risk predictivity.**

S. Table 1: 290 PD –associated SNPs from GWAS catalog to create model-1.

S. Table 2: Summary of CoDeS3D outputs for significant eQTLs for 290 PD-associated SNPs (Benjamini–Hochberg FDR < 0.05).

S. Table 3: David functional Annotation/Gene Ontology tables for the PD eGenes (n=1334).

S. Table 4: T1D SNPs (n=313) and PD GWAS SNPs (n=290) used in this study.

S. Table 5: PD Model-1 data features and model weights.

S. Table 6: PD Model-2 data features and model weights.

S. Table 7: PD risk predictive performance comparison of model-1 and model-2.

S. Table 8: GWAS catalog association for the PD SNPs (downloaded on 2020-08-27-Orphanet 2828-withChildTraits).

S. Table 9: Hi-C datasets used in this study.

S. Table 10: PD Illumina SNPs and individuals recommended for exclusion by the WTCCC.

S. Table 11: List of data features (eQTLs and SNPs) that were used in the modelling process.

S. Table 12: CoDeS3D output for sig eQTLs for the 313 T1D SNPs.
