# Supplementary material for: Machine Learning Identifies Six Genetic Variants and Alterations in the Heart Atrial Appendage as Key Contributors to PD Risk Predictivity
Source: Front Genet. 2022 Jan 3;12:785436. doi: 10.3389/fgene.2021.785436 (PMC8762216; doi:10.3389/fgene.2021.785436)
Supplement: Supplementary file 14 [file DataSheet1.docx]

**Supplementary Materials for Machine learning identifies genetic variants and changes in the Heart Atrial Appendage as making significant contributions to PD risk.**

**Supplementary Methods:**

**Tissue specific PD eQTL reference table**

GWAS SNPs associated with PD (n=290, *p-*value <1.0 x 10^-5^; Supplementary Table 1) were obtained from the GWAS catalogue (www.ebi.ac.uk/gwas, downloaded 27^th^ August 2020) (Supplementary Table 8). This SNP set included young adult-onset Parkinsonism SNPs(Siitonen et al., 2017) and the 90 SNPs identified by the most recent meta-analysis by *Nalls et al*.(Nalls et al., 2019).

The PD-associated SNPs (Supplementary Table 1) were analysed using the Contextualize Developmental SNPs in 3-Dimensions (CoDeS3D) algorithm(Farrow et al., 2021), with the beta effect calculation option, to identify: a) the genes that physically interact with the PD-associated SNPs; and b) which of these SNP-gene interactions are eQTLs. Physical interactions between PD-associated SNPs and genes were identified using Hi-C chromatin contact libraries (Supplementary Table 9) captured from:

1. Cell lines from primary human tissues (*e*.*g.* brain, skin and spinal cord)
2. Immortalised cell lines that represent the embryonic germ layers (*i.e*. HUVEC, NHEK, HeLa, HMEC, IMR90, KBM7, K562, and GM12878)

These libraries were chosen to represent tissues with the largest number of possible interactions that occur in the human system.

The potential regulatory effects (normalized effect size [NES]) of the spatial connections were mapped by leveraging the eQTL information from 49 human tissues (Genotype-Tissue Expression database [GTEx] v8; [www.gtexportal.org](http://www.gtexportal.org)). eQTL significance levels were adjusted for multiple testing [Benjamini–Hochberg FDR](Benjamini and Hochberg, 1995) and considered significant if *q*<0.05.

**WTCCC cohort cleaning and genotype imputation**

The PD genotype dataset was acquired from the WTCCC (Request ID 10584). The WTCCC PD genotype dataset, generated using Illumina microarrays, contained one case cohort (2197 individual samples) and two control cohorts (58C: 2930 individual samples and NBS: 2737 individual samples). Thus, the total number of control samples (n=5667) was more than double the number of cases. It is likely that the use of an imbalanced training dataset would create a biased disease status predictor. Therefore, only the control samples for the 58C 1958, British Birth cohort were used in this study.

SNPs and individual samples that were of poor quality and were recommended for study exclusion by the WTCCC were removed (Supplementary Table 10). SNPs within individual genotypes were converted to dbSNP rsIDs and genomic positions mapped (GRCh37, hg19) by Python scripts. PLINK (v1.90b6.2, 64-bit)(Purcell et al., 2007) was used for quality control. Genotypes were cleaned using the Method-of-moments F coefficient estimate to remove case homozygosity outliers (F values < -0.02 or 0.02 < F values) and the control outliers (F values < -0.016 or 0.19 < F values). Related individuals were identified and removed using proportion IBD (PI_HAT > 0.08). Ancestry outliers (identified by principal component analysis [PCA] plotting), individuals with sex genotype errors (identified by PLINK), or individuals with missing genotype data (missing rate > 5%) were also removed. Finally, SNPs that were significantly outside of Hardy-Weinberg Equilibrium (p < 10^-6^) or had a minor allele frequency < 1% were also removed.

The WTCCC PD case and control genotype data were obtained using two different Illumina microarrays (Human670-QuadCustom and Human1-2M-DuoCustom_v1_A)(Spencer et al., 2011). Therefore, we only used the 526,576 SNPs that were present in both microarrays for imputation. SNP data imputation was performed to recover a total of 27,590,399 SNPs using the Sanger imputation service (<https://imputation.sanger.ac.uk>), EAGLE+PBWT pipeline(Durbin, 2014; Loh et al., 2016), and Haplotype Reference Consortium(r1.1)(McCarthy et al., 2016). Imputation was performed according to the default instructions (https://imputation.sanger.ac.uk/?instructions=1). Following imputation, PLINK was used to update the genotype data with rsIDs and remove SNPs with an: impute2 score < 0.3; missing data rate > 5%; or those that were not in Hardy-Weinberg Equilibrium (p < 10^-6^). The genotypes for 281 of the 290 PD SNPs used in this study (Supplementary Table 1) were extracted from the imputed PD genotype data.

**The weighted WTCCC PD genotype eQTL effect matrix**

We created a matrix that combined individual genotypes with the eQTL effects for the PD-associated SNPs. There were three groups of data fields in the PD genotype eQTL table:

1. Individual sample information (sex, and disease status)
2. Individual sample PD-associated SNP genotype (SNP minor allele count) weighted by GTEx tissue-specific eQTL normalised effect sizes
3. Individual PD-associated SNP genotype for the SNPs with no eQTL effect information

The tissue-specific eQTL normalised effect size (NES) for the PD-associated SNPs were extracted from the GTEx eQTL summary table of significant eQTLs (Supplementary Table 2). The NES for each tissue-specific eQTL was weighted by the number of alternative alleles (0, 1 or 2) at the eQTL SNP position in each individual’s genome. 54 of the 290 PD-associated SNPs had no identifiable eQTL effects (Supplementary Table 11) and were input into the model unweighted, using solely SNP allele count from the imputed genotype.

We created two regularised logistic regression models (see below): for model-1, we created a weighted WTCCC PD genotype eQTL matrix for all 290 SNPs that were imputed and represented in the PD eQTL reference table. By contrast, for model-2 we created a weighted WTCCC PD genotype eQTL matrix for the subset of 90 SNPs from the PRS identified in *Nalls et al*.(Nalls et al., 2019).

**Training and validation of the regularised logistic regression models (model-1 and model-2)**

The weighted WTCCC PD genotype eQTL matrix that contained all the case and control genotypes that passed quality control (4366 individual samples: 1698 cases and 2668 controls) was used to train model-1 (using all 290 SNPs), or model-2 (using the 90 *Nalls et al.* SNPs).

The Mann-Whitney U test (tsfresh version 0.16.0)(Christ et al., 2018) was used to select the individual feature columns within the weighted WTCCC PD genotype eQTL matrix from the full training dataset that were the most relevant attributes for predicting PD status (*i.e*. the relevant attribute subset; FDR = 0.05)(Reiner et al., 2003). The relevant attribute subset was then used to train a multivariate logistic regression model (Scikit-learn version 0.23.2)(Abraham et al., 2014) implemented with elastic net regularisation using the SAGA solver to predict PD disease status. The machine learning elastic net regularisation prevented overfitting the predictor model by further sub-selecting the essential features for delivering the best prediction.

Training was optimised (measured by area under the receiver operating characteristic curve [AUC])(Vihinen, 2012) using a Scikit-learn Grid Search algorithm(Abraham et al., 2014; Mehta et al., 2018) with 10-fold cross-validation setting to select the optimised predictor model hyperparameters from the training stage (90% of cohort used for training, 10% used for cross validation). The optimised predictor hyperparameters for model-1 were: C=0.5, l1_ratio=0.6, max_iter=800, penalty='elasticnet‘, random_state=1, solver='saga‘ from the search space of following:

- 'C': 0.01, 0.05, 0.1, 0.5,1, 10, 20, 30,
- 'max_iter': 200, 500, 800, 1000, 1200, 1400, 1500, 1600,
- 'l1_ratio': 1, 0.9, 0.8, 0.7, 0.6, 0.5, 0.4, 0.3, 0.2, 0.1.

The optimised predictor hyperparameters for model-2 were: C=0.6, l1_ratio=0.1, max_iter=130, penalty='elasticnet‘, random_state=1, solver='saga‘ from the search of following:

- 'C': 0.0001, 0.001, 0.01, 0.1,0.2,0.3,0.4,0.5,0.6,0.7,0.8, 1, 3,
- 'max_iter': 1, 5, 70, 100, 130, 150, 170, 180, 200, 300, 500, 1000, 1200, 1400, 1600, 1800, 2000, 2200, 2400, 2600, 3000,
- 'l1_ratio': 1, 0.9, 0.8, 0.7, 0.6, 0.5, 0.4, 0.3, 0.2, 0.1.

The search space of models-1 and 2 did not include l1_ratio = 0 for excluding L2 regularisation and implementing feature selection. To calculate the variation in AUCs of the models with the optimised parameters we undertook 5 repeats of 10-fold cross-validation of model generation and validation by the Scikit-learn RepeatedKFold algorithm(Abraham et al., 2014). The 10-fold cross-validation started with the random generation of 10 equal parts from the full dataset. Nine parts of the data were used for training, and the remaining data were for validation. Mann Whitney U test filtering controlled by FDR = 0.05 was applied to the training set. Subsequently, the filtered training data were modelled by the multiple regularised logistic regression algorithm with the optimised predictor hyperparameters of (model-1 or model-2). This process was repeated until all parts of the data were used for validation. The result of this process is the final PD predictor for each model, with in-sample (training data) predictive performance as assessed by AUC.

**UK Biobank cohort definition**

Genotypes (case and control) that were used from the UK Biobank were selected as follows. European Caucasian samples identified by genetic clustering methods were selected and imputed (487,411 individual samples). The genomic relatedness analysis excluded SNPs that the UK Biobank recommended were removed from the selected case and control data.

The cases (model-1: 928 cases or model-2:1484 cases) were selected using the following criteria:

1. PD patient identified by the UK Biobank developed algorithm (field 42033)
2. PD patient identified by hospital records G20
3. PD patient had no missing data for any SNPs within the predictor model (model-1 or model-2). The greater number of SNPs used in model-1 meant that more cases were excluded due to missing data.

Control genotypes, not having records of Parkinsonism and without missing data for any of the SNPs included in the final predictor, were randomly selected from the healthy controls within the UK Biobank data for each of the 30 test cohorts. As model 2 had more cases, more controls were also included so as to match the ratio of case:control.

Genotype data of the UK Biobank case and control samples in each test cohort were used to build a weighted eQTL-genotype matrix for testing model-1 or 2 to recognise the disease status of individual samples correctly.

**NeuroX-dbGap cohort definition**

Genotypes were also obtained from the NeuroX-dbGap dataset. Genotypes were cleaned by removing all insertion and deletion variants. SNP IDs were converted to dbSNP rsIDs. Variants in chromosome 24(Y), 25(XY) and 26(MT) that are not included in the study due to the inconsistency with the Sanger imputed SNP data were also removed. Ancestry outliers (identified by principal component analysis [PCA] plotting), individuals with sex genotype errors (identified by PLINK), or individuals with missing genotype data (missing rate > 5%) were also removed. Finally, SNPs that were not in Hardy-Weinberg Equilibrium (p < 10^-5^) or had a minor allele frequency < 1% were removed. All the variants in the final model (model-1 or model-2) which were not present in the NeuroX-dbGap data were replaced with proxy SNPs using linkage disequilibrium information (r^2^> 0.5)(Nalls et al., 2015) calculated by PLINK from European 1000 genome genotype data (<https://www.internationalgenome.org/about>)(Auton et al., 2015). The European 1000 genome genotype data were downloaded from (<https://ctg.cncr.nl/software/magma>)(de Leeuw et al., 2015) on 10^th^ August 2020.

**Mann-Whitney U test filtering on 290 PD and 313 T1D SNPs derived eQTL matrix**

We generated a GTEx eQTL summary table of significant eQTLs (Supplementary Table 12) using 313 Type 1 Diabetes (T1D) associated SNPs (Supplementary Table 4). We mixed the 290 PD and 313 T1D SNPs to create a weighted WTCCC PD and T1D genotype eQTL matrix, as outlined above. Mann-Whitney U test filtering (controlled by FDR = 0.05) was applied to the weighted WTCCC PD and T1D genotype eQTL matrix to determine the filtering power for removing non-related PD features.

**Supplementary Figures:**

**
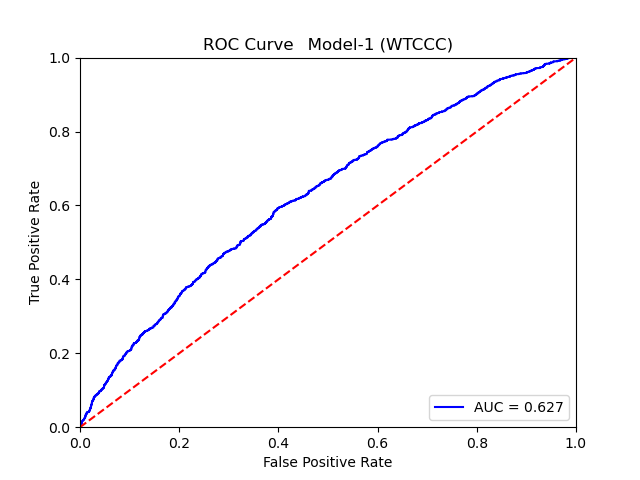
**

**Supplementary Figure 1:** ROC Curve of Model-1 on WTCCC derived genotype eQTL effect matrix


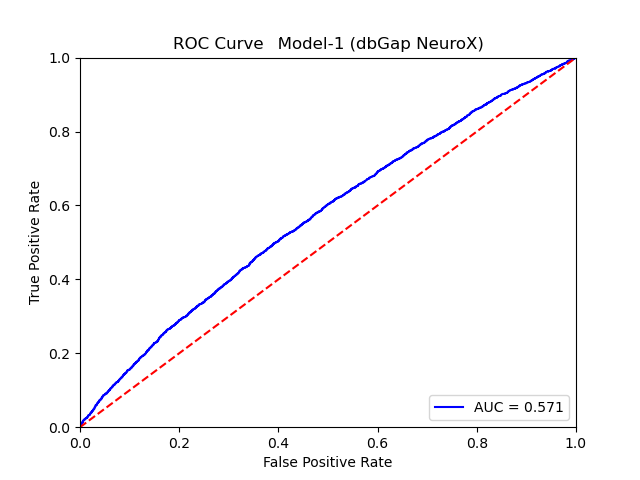


**Supplementary Figure 2:** ROC Curve of Model-1 on NeuroX derived genotype eQTL effect matrix


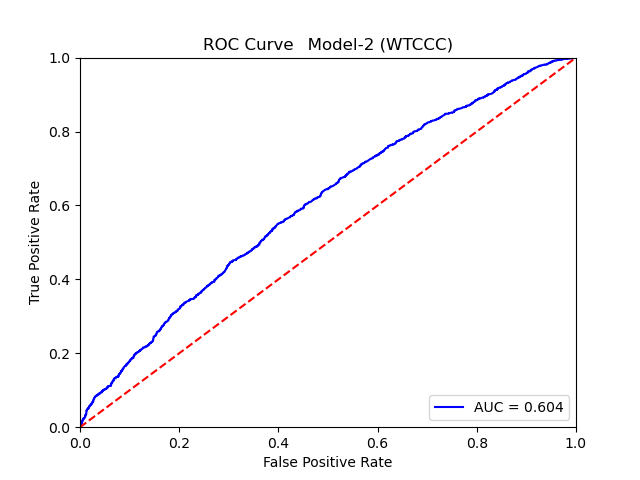


**Supplementary Figure 3:** ROC Curve of Model-2 on WTCCC derived genotype eQTL effect matrix


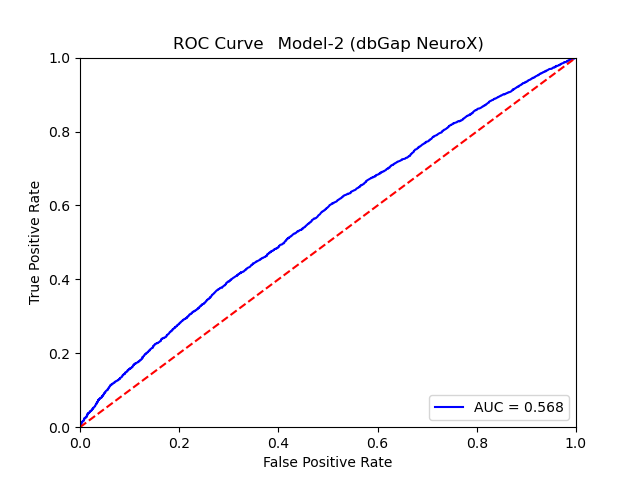


**Supplementary Figure 4:** ROC Curve of Model-2 on NeuroX derived genotype eQTL effect matrix

**References:**

Abraham, A., Pedregosa, F., Eickenberg, M., Gervais, P., Mueller, A., Kossaifi, J., et al. (2014). Machine learning for neuroimaging with scikit-learn. *Front. Neuroinform.* 8, 14.

Auton, A., Abecasis, G. R., Altshuler, D. M., Durbin, R. M., Bentley, D. R., Chakravarti, A., et al. (2015). A global reference for human genetic variation. *Nature* 526, 68–74. doi:10.1038/nature15393.

Benjamini, Y., and Hochberg, Y. (1995). Controlling the false discovery rate: a practical and powerful approach to multiple testing. *J. R. Stat. Soc. B* 57, 289–300. doi:10.2307/2346101.

Christ, M., Braun, N., Neuffer, J., and Kempa-Liehr, A. W. (2018). Time Series FeatuRe Extraction on basis of Scalable Hypothesis tests (tsfresh – A Python package). *Neurocomputing* 307, 72–77. doi:10.1016/j.neucom.2018.03.067.

de Leeuw, C. A., Mooij, J. M., Heskes, T., and Posthuma, D. (2015). MAGMA: Generalized Gene-Set Analysis of GWAS Data. *PLoS Comput. Biol.* 11, 1–19. doi:10.1371/journal.pcbi.1004219.

Durbin, R. (2014). Efficient haplotype matching and storage using the positional Burrows-Wheeler transform (PBWT). *Bioinformatics* 30, 1266–1272. doi:10.1093/bioinformatics/btu014.

Farrow, S. L., Schierding, W., Gokuladhas, S., Golovina, E., Fadason, T. M., Cooper, A. A., et al. (2021). Establishing gene regulatory networks from Parkinson’s disease risk loci. *bioRxiv*, 2021.04.08.439080. doi:10.1101/2021.04.08.439080.

Loh, P.-R., Danecek, P., Palamara, P. F., Fuchsberger, C., A Reshef, Y., K Finucane, H., et al. (2016). Reference-based phasing using the Haplotype Reference Consortium panel. *Nat. Genet.* 48, 1443–1448. doi:10.1038/ng.3679.

McCarthy, S., Das, S., Kretzschmar, W., Delaneau, O., Wood, A. R., Teumer, A., et al. (2016). A reference panel of 64,976 haplotypes for genotype imputation. *Nat. Genet.* 48, 1279.

Mehta, P., Bukov, M., Wang, C.-H., Day, A. G. R., Richardson, C., Fisher, C. K., et al. (2018). A high-bias, low-variance introduction to Machine Learning for physicists. *Phys. Rep.* doi:10.1016/B978-0-08-044132-0.50011-3.

Nalls, M. A., Blauwendraat, C., Vallerga, C. L., Heilbron, K., Bandres-Ciga, S., Chang, D., et al. (2019). Identification of novel risk loci, causal insights, and heritable risk for Parkinson’s disease: a meta-analysis of genome-wide association studies. *Lancet Neurol.* 18, 1091–1102. doi:10.1016/S1474-4422(19)30320-5.

Nalls, M. A., Bras, J., Hernandez, D. G., Keller, M. F., Majounie, E., Renton, A. E., et al. (2015). NeuroX, a fast and efficient genotyping platform for investigation of neurodegenerative diseases. *Neurobiol. Aging* 36, 1605.e7-1605.e12. doi:10.1016/j.neurobiolaging.2014.07.028.

Purcell, S., Neale, B., Todd-Brown, K., Thomas, L., Ferreira, M. A. R., Bender, D., et al. (2007). PLINK: a tool set for whole-genome association and population-based linkage analyses. *Am. J. Hum. Genet.* 81, 559–575.

Reiner, A., Yekutieli, D., and Benjamini, Y. (2003). Identifying differentially expressed genes using false discovery rate controlling procedures. *Bioinformatics* 19, 368–375. doi:10.1093/bioinformatics/btf877.

Siitonen, A., Nalls, M. A., Hernández, D., Gibbs, J. R., Ding, J., Ylikotila, P., et al. (2017). Genetics of early-onset Parkinson’s disease in Finland: exome sequencing and genome-wide association study. *Neurobiol. Aging* 53, 195-e7.

Spencer, C. C. A., Plagnol, V., Strange, A., Gardner, M., Paisan-Ruiz, C., Band, G., et al. (2011). Dissection of the genetics of Parkinson’s disease identifies an additional association 5’ of SNCA and multiple associated haplotypes at 17q21. *Hum. Mol. Genet.* 20, 345–353. doi:10.1093/hmg/ddq469.

Vihinen, M. (2012). How to evaluate performance of prediction methods? Measures and their interpretation in variation effect analysis. *BMC Genomics* 13 Suppl 4. doi:10.1186/1471-2164-13-S4-S2.
